# Impact of Regular Yoga and Meditation on Immune Cell Populations: A Small-Sample, Single-Arm, Pre-Post Interventional Pilot Study

**DOI:** 10.7759/cureus.107488

**Published:** 2026-04-21

**Authors:** Luis Francisco Castillo-Torres, Selene Velázquez-Moreno, Carla Edith Tovar-Leyva, Carolina Reyes-Martínez, Rita Elizabeth Martínez-Martínez, Carolina Rivera-Luque, Marlen Vitales-Noyola

**Affiliations:** 1 Department of Holistic Therapy, Shanti Peace House, San Luis Potosí, MEX; 2 Faculty of Dentistry, Autonomous University of San Luis Potosi, San Luis Potosí, MEX; 3 Faculty of Chemistry, Autonomous University of San Luis Potosi, San Luis Potosí, MEX; 4 Department of Health and Sport Sciences, Technical University of Munich (TUM) School of Medicine and Health, Munich, DEU

**Keywords:** cd14, cd19, cd4, cd8 cells, immune cells, yoga and meditation, yoga therapy, lymphocytes

## Abstract

Background

Immunity is the body's ability to resist infectious diseases and is regulated by the immune system, which is divided into innate immunity, with an immediate response, and adaptive immunity, which is more specialized and effective. Lymphocytes, such as CD4^+^, CD8^+^, and CD19^+^, are part of adaptive immunity, while monocytes/macrophages, CD14^+^, are phagocytic cells of the innate immune system and maintain homeostasis.

Objectives

The objective of this study was to evaluate the effect of a short-term yoga and meditation intervention on specific immune cell populations (CD4+, CD8+, CD19+, and CD14+) in healthy adults practicing yoga, using a pilot pre-post study design.

Methodology

A prospective pre-post pilot study was conducted in five healthy adult participants who were regular practitioners of yoga and meditation and attended a 15-day meditation retreat. Peripheral blood samples were collected before and after the intervention. Immune cell populations, including CD4+ T lymphocytes, CD8+ T lymphocytes, CD19+ B cells, and CD14+ monocytes, were quantified using flow cytometry following standard Ficoll-based peripheral blood mononuclear cell isolation. Data normality was assessed using the Shapiro-Wilk test, and paired statistical analyses were performed to evaluate changes between pre- and post-intervention measurements.

Results

An increase in immune cell populations, including CD4+ T lymphocytes, CD8+ T lymphocytes, CD19+ B cells, and CD14+ monocytes, was observed following the intervention compared to baseline, with statistically significant differences (p<0.05).

Conclusions

Short-term yoga and meditation practice were associated with increases in adaptive and innate immune cell populations in this pilot study. These results suggest a potential link between regular practice and immune response, but larger controlled studies are required to draw definitive conclusions.

## Introduction

Immunity refers to the body’s capacity to resist disease, particularly those caused by infectious agents. The collection of cells, tissues, and molecular components responsible for this protective function constitutes the immune system, while the integrated actions of these elements against invading microorganisms are known as the immune response. Host defense mechanisms consist of innate immunity, which mediates the initial protection against infections, and adaptive immunity, which develops more slowly and provides a more specialized and effective defense against infections [1]. The adaptive immune system is composed of lymphocytes and their products, including antibodies, while innate immune defense against intracellular viruses is mainly mediated by natural cytolytic lymphocytes [1,2].

Among the key cellular components of the adaptive immune system are mainly CD4⁺ T lymphocytes, also known as helper T cells. These cells play a central role in regulating immune responses by activating other immune cells, including B lymphocytes, cytotoxic T lymphocytes, and macrophages, through the secretion of cytokines [3]. CD8⁺ T lymphocytes, or cytotoxic T cells, are crucial for the direct elimination of virus-infected or malignantly transformed cells through the release of perforins and granzymes [4]. CD19⁺ B lymphocytes are responsible for humoral immunity; they recognize specific antigens and differentiate into plasma cells that produce antigen-specific antibodies, playing an essential role in neutralizing pathogens and facilitating their clearance through opsonization and complement activation [5]. On the other hand, CD14⁺ monocytes and macrophages are part of the innate immune system. They act as antigen-presenting cells and are critical for phagocytosis of pathogens and cellular debris. Additionally, they produce pro-inflammatory cytokines that shape the early immune response and influence the adaptive response by presenting processed antigens to T cells [5]. The dynamic balance and functionality of these immune cell subsets are essential for maintaining immune homeostasis and an effective defense against pathogens [5,6].

Emerging evidence suggests that lifestyle interventions such as regular yoga and meditation may modulate the immune system by influencing the distribution, activation, and functional profile of specific immune cell subpopulations, including CD4⁺ and CD8⁺ T lymphocytes, CD19⁺ B lymphocytes, and CD14⁺ monocytes/macrophages [7,8]. This immunomodulatory potential is gaining increasing attention, particularly in the context of psychoneuroimmunology, a field that explores the dynamic interactions between psychological processes, the nervous system, and immune regulation [8]. In recent years, numerous studies have explored the complex and bidirectional relationship between the mind and the immune system [9-11]. Chronic psychological stress is recognized as a significant contributor to immune dysregulation, leading to elevated levels of pro-inflammatory cytokines and suppression of cellular immunity [11]. These immune alterations have been linked to increased susceptibility to infections, delayed wound healing, and the progression of chronic inflammatory diseases. As a result, the widespread prevalence of stress has prompted growing interest in integrative approaches to stress management, including structured practices such as yoga and meditation [12].

Yoga, as an integrative mind-body practice, incorporates physical poses (asanas), regulated breathing methods (pranayama), and meditation techniques (*dhyana*). There are many types of yoga, including hatha, vinyasa, kundalini, yin, ashtanga, bikram, restorative, jivamukti, anusara, bhakti, and acroyoga [12]. The positive benefits of yoga therapies are achieved in part by reducing inflammation and improving various immune parameters of the immune response. This multifaceted approach has demonstrated efficacy in reducing perceived stress, anxiety, and depression, while simultaneously promoting emotional resilience and psychological well-being [13]. Physiologically, regular yoga practice has been associated with reductions in cortisol levels, sympathetic nervous system activity, blood pressure, and blood glucose, all of which contribute to a more balanced neuroendocrine and immune state [14]. Moreover, recent clinical and experimental studies have reported that yoga and meditation may lead to favorable changes in immune biomarkers, including increased proportions of naïve and memory T cells, enhanced natural killer (NK) cell activity, and modulation of inflammatory cytokine profiles [15]. A recent meta-analysis examining the impact of yoga on stress and immunity concluded that yoga interventions can significantly lower markers of systemic inflammation (e.g., interleukin 6 (IL-6), tumor necrosis factor-alpha (TNF-α)) and improve immune surveillance, suggesting a potential protective role in both healthy individuals and those with immune-related conditions [16].

Taken together, these findings support the hypothesis that regular yoga and meditation practice may be associated with changes in specific immune cell populations (CD4+, CD8+, CD19+, and CD14+) and could contribute to the modulation of the immune response in healthy adults. In this pilot study, we evaluated immune cell populations in healthy individuals who regularly practiced yoga and meditation and who participated in a 15-day immersive retreat focused on these disciplines, aiming to explore potential immunological changes associated with sustained mind-body interventions.

## Materials and methods

This study was designed as a small-sample, single-arm, pre-post pilot interventional study to evaluate the effects of regular yoga and meditation practice on immune cell populations in healthy adult participants. The study was conducted at the Faculty of Dentistry, Autonomous University of San Luis Potosí, San Luis Potosí, Mexico. 

The study was carried out in compliance with the ethical standards outlined in the Declaration of Helsinki for studies involving human participants and was approved by the Ethics and Research Committee of the Faculty of Stomatology, Autonomous University of San Luis Potosi (approval number: CEIFE-078-024).

Study population

Five healthy adult volunteers were recruited from the Wellness Center of Shanti Peace House, located in San Luis Potosí, Mexico. All participants were over 18 years of age and engaged in regular yoga and meditation practice. The pilot study was conducted during a 15-day retreat held in a secluded location, offering a tranquil natural environment conducive to silence, introspection, and immersive mind-body practices. All participants were apparently healthy and signed an informed consent form after being informed about the retreat activities, location, and the procedures related to study processes.

Study process

Blood Sample Collection

A 4 mL peripheral blood sample was collected from each participant into ethylenediaminetetraacetic acid (EDTA)-coated vacutainer tubes one to two hours prior to the start of the retreat. A second blood sample was obtained immediately upon participants’ return from the 15-day retreat. Peripheral blood mononuclear cells (PBMCs) were separated by density-gradient centrifugation using Ficoll-Hypaque (GE Healthcare Technologies, Inc., Chicago, Illinois, United States). Cell viability was assessed by trypan blue exclusion staining and consistently exceeded 95%.

Retreat Activities

The daily program in the retreat focused on meditation, rest, and a vegan diet, following a structured schedule detailed in Table 1.

**Table 1:** Retreat schedule and daily activities

Time	Activity
21:30 – 04:00	Sleep time
04:00 – 04:30	Wake up
04:30 – 06:00	Meditation (hall or bedroom)
06:00 – 08:00	Breakfast and rest
08:00 – 09:00	Group meditation (hall)
09:00 – 11:00	Meditation (hall or bedroom)
11:00 – 12:00	Lunch
12:00 – 13:00	Rest and guide interviews
13:00 – 14:30	Meditation (hall or bedroom)
14:30 – 15:30	Group meditation (hall)
15:30 – 17:00	Meditation (hall or bedroom)
17:00 – 18:00	Snack and rest
18:00 – 19:00	Group meditation (hall)
19:00 – 20:15	Guide’s talk
20:15 – 21:00	Group meditation (hall)
21:00 – 21:30	Q\&A session in the hall

The meditation practice varied throughout the retreat, progressing through *Anapanasati*, *Vipassana*, and *Metta Bhavana* techniques, as outlined in Table 2

**Table 2:** Meditation techniques practiced during the retreat and their objectives

Retreat Day(s)	Meditation Technique	Purpose
First to third day	Anapanasati	Respiratory focus
Fourth to ninth day	Vipassana	Observation of body sensations, emotions, and thoughts
Tenth to fifteenth day	Vipassana + Metta bhavana	Cultivation of empathy, positivity, acceptance, and compassion towards oneself and others

Flow Cytometry Analysis

Mononuclear cells were incubated for 30 minutes at 4 °C in the dark with the following monoclonal antibodies (mAbs): CD4-PerCP (eBioscience, Inc., San Diego, California, United States), CD8-APC (BioLegend, San Diego, California, United States), CD19-PE (Becton, Dickinson and Company, Franklin Lakes, New Jersey, United States), and CD14-FITC (eBioscience, Inc.). Doublets were excluded by evaluating FSC-A versus FSC-W dot plots within the lymphocyte gate. In all samples, a minimum of 1 × 10⁶ events was acquired, and gating strategies were established using fluorescence-minus-one (FMO) controls together with isotype-matched monoclonal antibody controls. Data were acquired in an Attune flow cytometer (Thermo Fisher Scientific Inc., Waltham, Massachusetts, United States) and analyzed using the Flow Jo software v.10.0 (Tree Star Inc., Ashland, Orlando, United States).

Statistical analysis

Continuous variables are presented as mean ± standard deviation (SD) for normally distributed data and as median with interquartile range (IQR) (Q1-Q3) for non-normal distributions. Data normality was assessed using the Shapiro-Wilk test, which is recommended for small sample sizes (n < 30), to determine whether parametric or non-parametric tests should be applied. Comparisons between two time points (pre- vs post-intervention) were performed using a paired Student’s t-test for normally distributed data or the Wilcoxon signed-rank test for non-normal data. At the end of the study, differences among the four immune cell populations (CD4+, CD8+, CD19+, and CD14+) were assessed using one-way ANOVA for normally distributed data or the Kruskal-Wallis test for non-normal data, followed by suitable post hoc analyses. All statistical analyses were performed using GraphPad Prism version 5.0 (Dotmatics, Boston, Massachusetts, United States), and a p-value < 0.05 was considered statistically significant.

## Results

Participant demographics and baseline clinical characteristics

A total of five participants were included in the study. The clinical and demographic data of the participants are summarized in Table 3. This includes information such as age, sex, and relevant health and lifestyle characteristics related to yoga and meditation practice.

**Table 3:** Clinical and demographic data from participants *including chronic, autoimmune, degenerative, metabolic diseases or recent infections, or habitual use of drugs **Total values are given as percentages and mean±SD F: female, M: male; SD: standard deviation; V: vinyasa; A: ashtanga

Participant	Gender (F/M)	Age (years)	Weight (kg)	Heigth (cm)	BMI (kg/m^2^)	Diseases*	Style of yoga practiced	Frequency of practice (days per week)	Previous yoga and meditation retreat (yes/no)
1	F	28	70	163	26.3	No	Vinyasa	2	Yes
2	M	38	85	180	26.2	No	Ashtanga	3-4	Yes
3	F	41	45	154	19	No	Ashtanga	3-4	No
4	F	40	61	161	23.5	No	Ashtanga	5-7	Yes
5	M	31	60	169	21	No	Ashtanga	5-7	Yes
Total**	F=60%; M=40%	35.6±5.8	64.2±14.6	165.4±9.7	23.2±3.2	-	V=20%; A=80%	-	-

Levels of immune cellular subpopulation before and after the retreat

**Figure 1:**
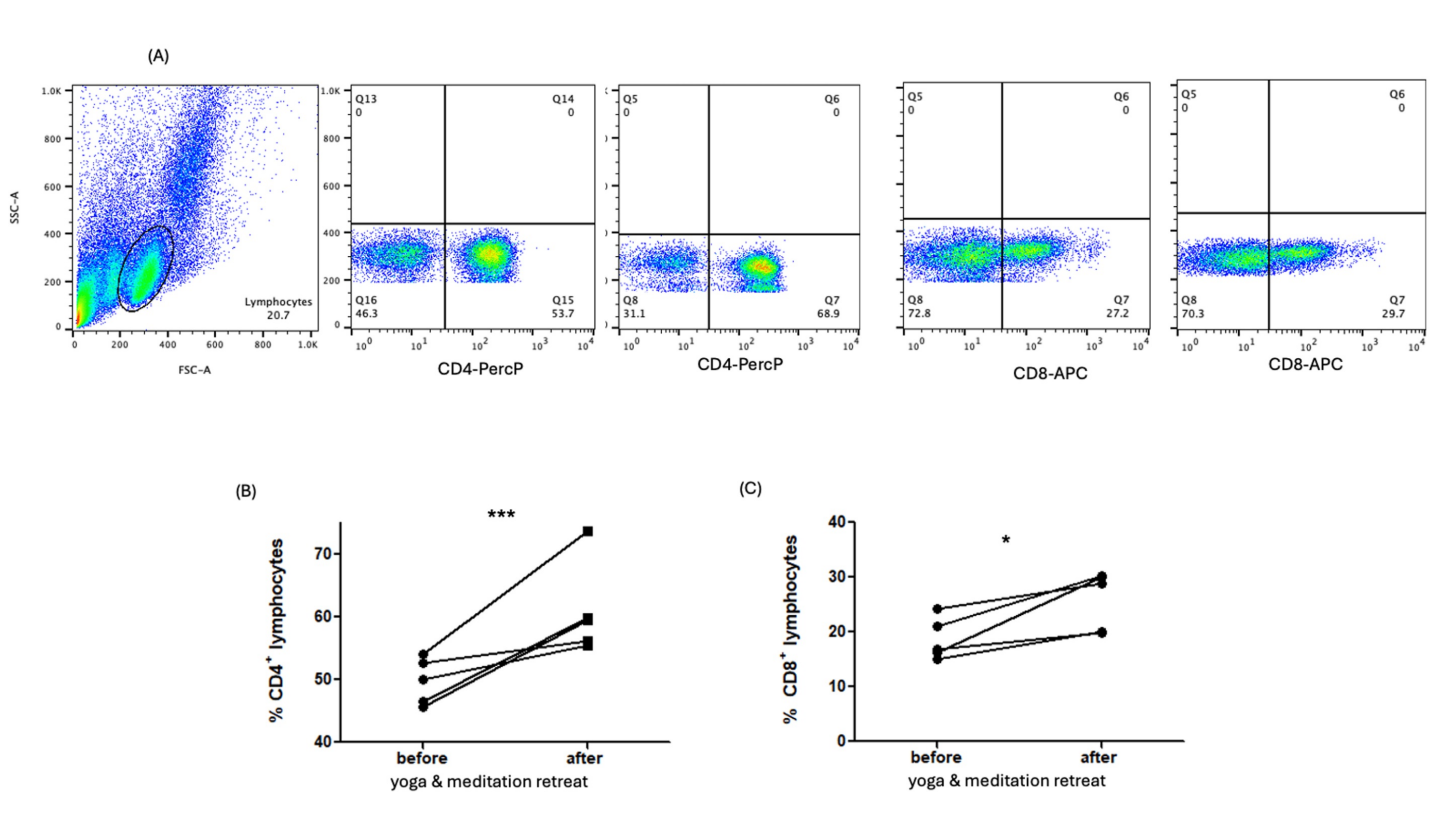
Representative flow cytometry dot plots and quantification of T lymphocyte populations before and after the 15-day yoga and meditation retreat (N=5) (A) Representative dot plots showing gating strategy and distribution of CD4+ and CD8+ T cells. (B) Scatter plot depicting the percentage of CD4+ T lymphocytes pre- and post-retreat. (C) Scatter plot depicting the percentage of CD8+ T lymphocytes pre- and post-retreat. *statistically significant difference (p < 0.05) between groups.

Multiple immune cell subpopulations were assessed by flow cytometry (Figure 1A). A significant increase was observed in the proportion of CD4+ T lymphocytes following the 15-day yoga and meditation retreat. Specifically, the percentage of CD4+ cells increased from a median 50.0 (IQR 46.09-53.3%) to 59.49 (IQR 55.81-70%), after the retreat (p=0.0079) (Figure 1B). Similarly, the percentage of CD8+ T cells also increased significantly, from 20.38±6.28%, mean and standard deviation, respectively, at 26.24±5.93% post-retreat (p=0.0446) (Figure 1C).

**Figure 2:**
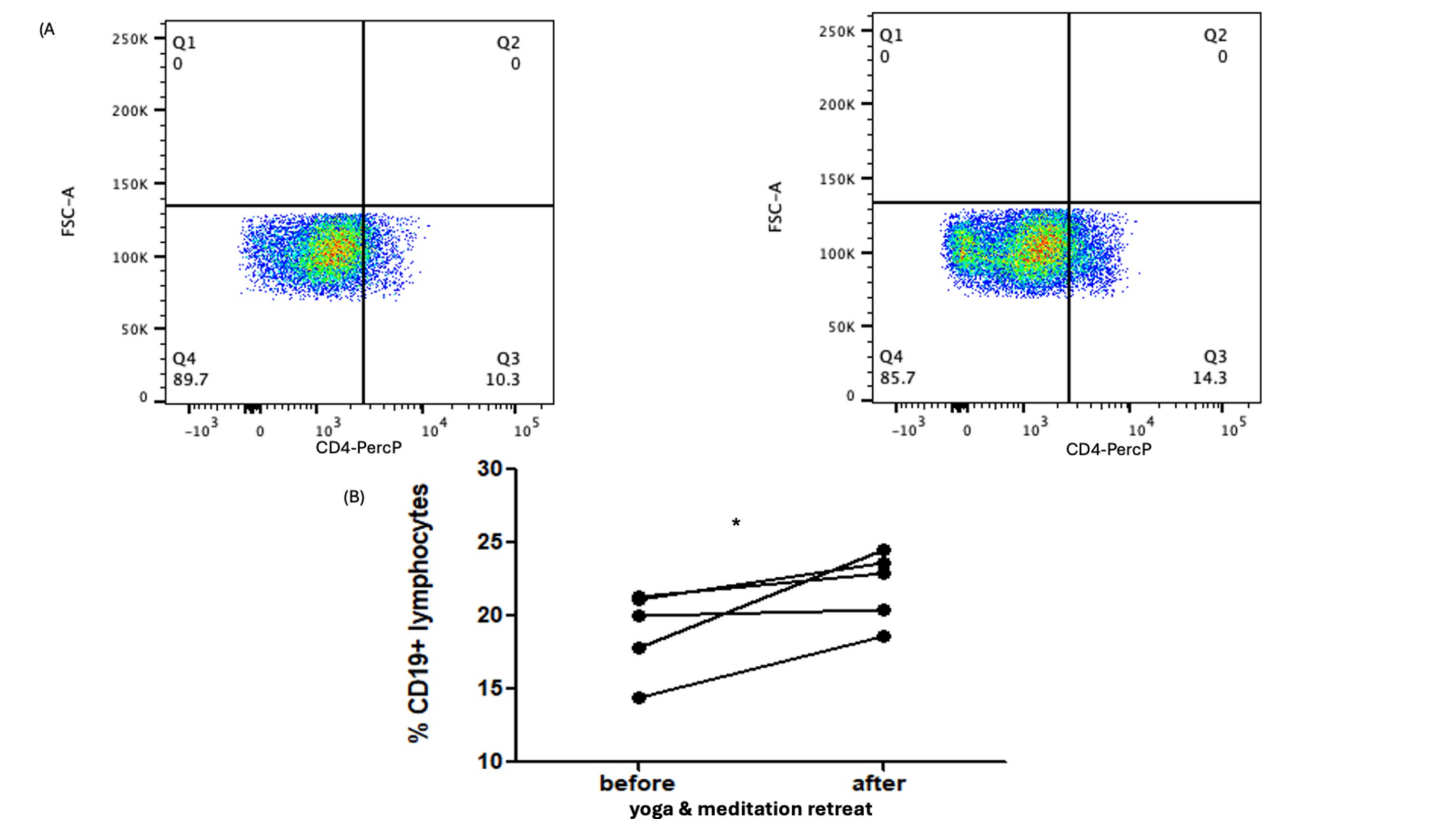
Representative flow cytometry dot plots and quantification of B lymphocytes before and after the 15-day yoga and meditation retreat (N=5) (A) Representative dot plots showing gating and distribution of CD19+ B cells. (B) Scatter plot illustrating the percentage of CD19+ B lymphocytes pre- and post-retreat. *statistically significant difference (p < 0.05) between groups

Notably, a significant increase was also detected in CD19+ B lymphocytes, with values rising from 18.92±2.89% to 22.0±2.457% post retreat (p=0.0477) (Figures 2A, 2B)

**Figure 3:**
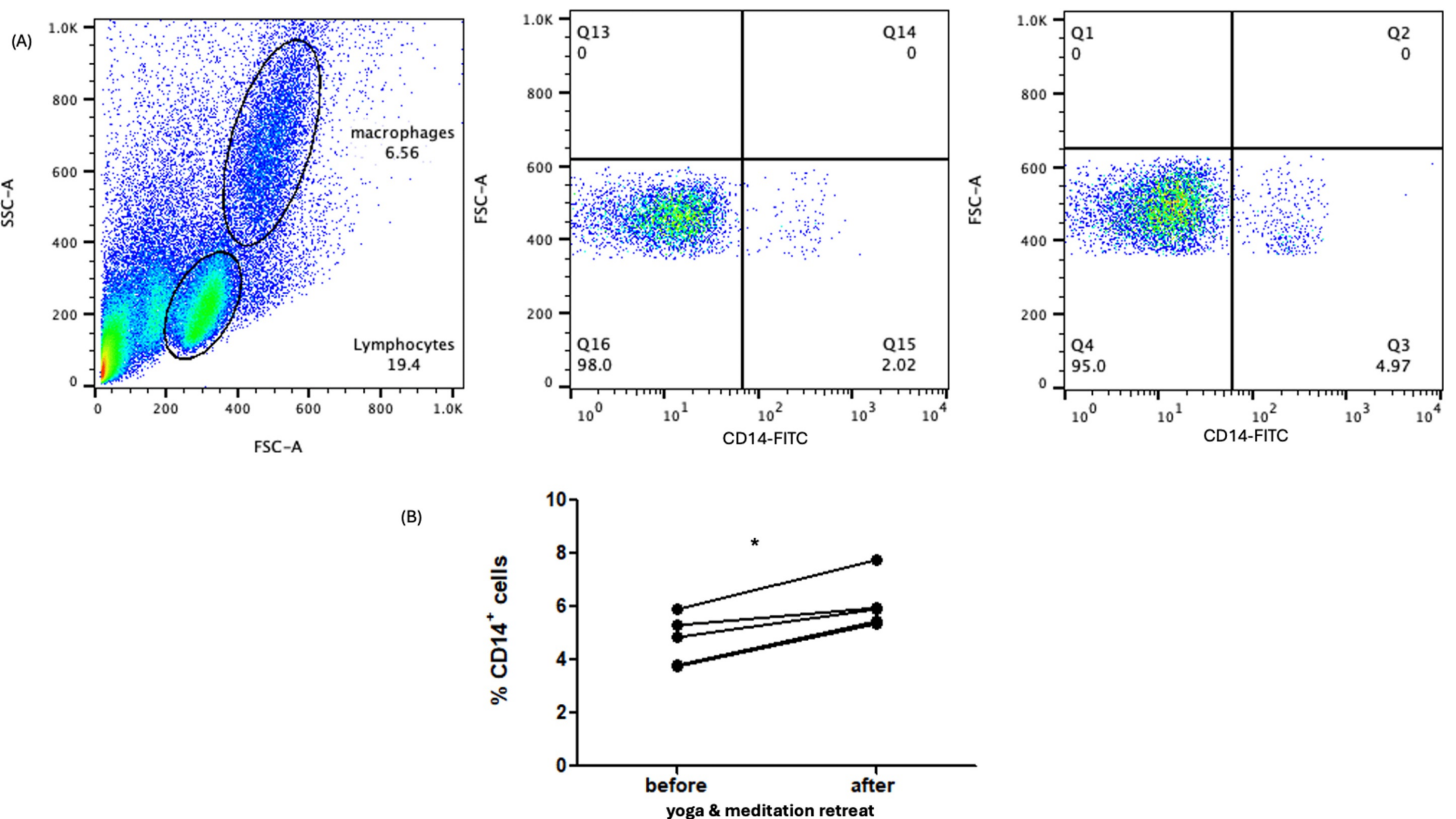
Representative flow cytometry dot plots and quantification of monocytes before and after the 15-day yoga and meditation retreat (N=5) (A) Representative dot plots showing gating and distribution of CD14+ monocytes. (B) Scatter plot illustrating the percentage of CD14+ monocytes pre- and post-retreat. *statistically significant difference (p < 0.05) between groups

Interestingly, CD14+ monocytes also exhibited a statistically significant rise, increasing from a median of 4.87 (IQR 3.76-5.607%) to 5.909 (IQR 5.39-6.856%) after the retreat (p=0.05) (Figures 3A, 3B).

Percentage of increase in each cellular subpopulation

**Figure 4:**
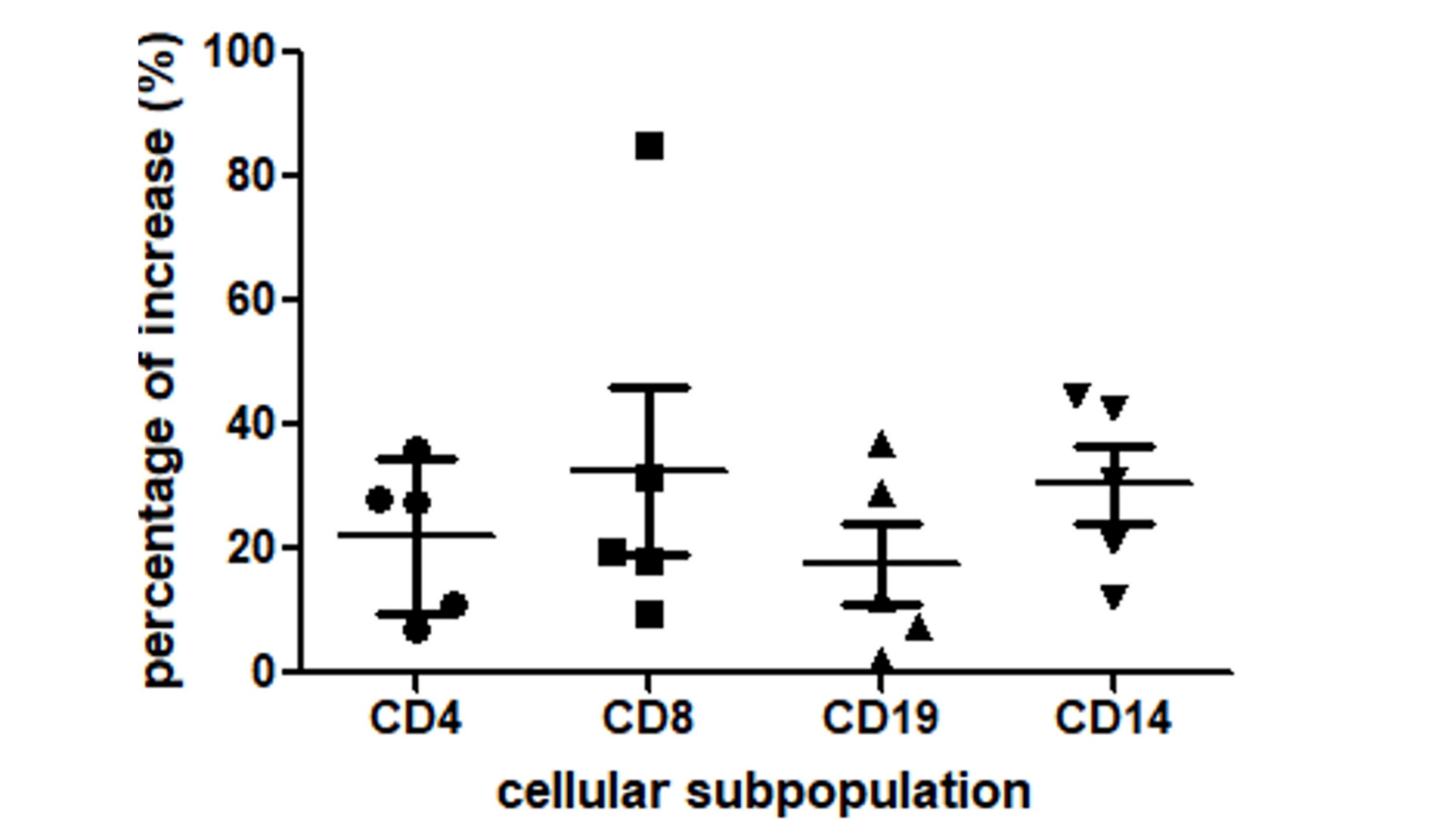
Percentage increase in immune cell populations for each participant following the 15-day yoga and meditation retreat (N=5) The graph shows individual changes in CD4+ T cells, CD8+ T cells, CD19+ B cells, and CD14+ monocytes expressed as the percentage increase from baseline.

In addition, the percentage of increase in each subcellular population was evaluated from participants. All subpopulations showed post-intervention increases (CD4⁺, CD8⁺, CD19⁺, CD14⁺) (Figure 4).

## Discussion

The immune system is essential for preserving health, as it protects the body from infectious agents and modulates inflammatory processes [17]. However, chronic psychological stress and lifestyle factors can negatively impact immune function, leading to increased susceptibility to illness [18]. In recent years, mind-body interventions, including yoga and meditation, have attracted increasing scientific interest for their potential to modulate immune responses and improve overall well-being [19].

This pilot study observed that participation in a 15-day yoga and meditation retreat was associated with increases in multiple immune cell subpopulations, including CD4+ and CD8+ T lymphocytes, CD19+ B cells, and CD14+ monocytes. While these findings suggest a potential modulation of both adaptive and innate immune responses following short-term practice of contemplative disciplines, causal conclusions cannot be drawn due to the small sample size, absence of a control group, and presence of multiple uncontrolled variables such as diet, environmental conditions, and stress reduction.

The observed increase in CD4+ T lymphocytes may reflect a shift toward greater immunosurveillance and immune regulation, potentially mediated by reduced psychological stress. CD4+ lymphocytes, especially the Th1 and Th2 subsets, play a central role in coordinating immune responses, and prior studies have shown that psychological stress reduction through meditation is associated with improved T cell function [20]. Similarly, the rise in CD8+ T cells (or cytotoxic lymphocytes), which are key effectors in viral and tumor immunity, supports the notion that continuous practice of yoga and meditation may enhance cytotoxic responses. Although evidence on CD8+ modulation is less abundant, a few studies have shown increased CD8+ cells after mindfulness or pranayama-based interventions [21]. The significant increase in CD19+ B cells observed in our study aligns with reports suggesting that mind-body practices can upregulate humoral immune activity. B lymphocytes are responsible for antibody production, and their expansion may indicate improved readiness of the immune system to respond to pathogens. A study by Gopal et al. in 2012 reported similar increases in antibody titers following mindfulness training [22]. On the other hand, the increase in CD14+ monocytes is particularly notable, as these innate immune cells are early responders to inflammatory signals, among other functions. Some studies have found that yoga and meditation can reduce circulating inflammatory cytokines such as IL-6 and TNF-α, suggesting a more regulated monocyte response rather than chronic activation [23-26]. The findings might indicate a mobilization of monocytes toward tissue surveillance and repair processes in the absence of systemic inflammation.

Previous studies have demonstrated that even a 90-minute stretching exercise can significantly increase levels of human β-defensin 2, an important antimicrobial peptide involved in innate immunity [24]. This supports the notion that physical components of yoga may directly enhance immune defenses. Moreover, yoga practice has been reported to inhibit the decline in cellular immunity commonly associated with psychological stress, thereby helping to maintain immune homeostasis [25]. These effects likely contribute to the immunomodulatory benefits observed in our study, where mind-body interventions appear to counteract stress-induced immune suppression and promote a more robust immune profile. Taken together, these immunological changes suggest that integrated yoga and meditation practices may promote a state of enhanced immune readiness without triggering pathological activation. This aligns with the concept of immune resilience, where the immune system is more responsive to threats yet remains tightly regulated. The underlying neuroendocrine mechanisms likely include regulation of the hypothalamic-pituitary-adrenal (HPA) axis and a decrease in cortisol secretion and increased parasympathetic activity, factors known to influence immune function [26].

While our study provides encouraging evidence of yoga and meditation-induced immune enhancement, limitations include the absence of a control group, the short follow-up period, and the lack of pro- and anti-inflammatory cytokines or stress hormone measurements. In addition, the small sample size limits the generalizability of our findings, and the results should be interpreted with caution. This study should therefore be considered a pilot investigation, aimed at generating hypotheses and guiding future research with larger, controlled cohorts. Future studies should also explore the long-term sustainability of these effects and their clinical relevance, particularly in populations with immune dysregulation.

## Conclusions

The findings of this pilot study indicate that short-term participation in a yoga and meditation retreat was associated with increases in multiple immune cell populations, including helper and cytotoxic T cells, B cells, and monocytes. These observations suggest a potential modulation of both adaptive and innate immune responses; however, causal relationships cannot be established due to the small sample size, absence of a control group, and the presence of multiple uncontrolled variables such as diet, environment, and stress reduction. Overall, the results provide preliminary evidence of a possible association between regular yoga and meditation practice and changes in immune cell populations. Further studies with larger sample sizes and controlled designs are needed to confirm these findings and explore their potential clinical relevance.
